# MuWU: Mutant-seq library analysis and annotation

**DOI:** 10.1093/bioinformatics/btab679

**Published:** 2021-09-29

**Authors:** Tyll Stöcker, Lena Altrogge, Caroline Marcon, Yan Naing Win, Frank Hochholdinger, Heiko Schoof

**Affiliations:** Crop Bioinformatics, Institute of Crop Science and Resource Conservation (INRES), University of Bonn, 53115 Bonn, Germany; Crop Bioinformatics, Institute of Crop Science and Resource Conservation (INRES), University of Bonn, 53115 Bonn, Germany; Crop Functional Genomics, Institute of Crop Science and Resource Conservation (INRES), University of Bonn, 53115 Bonn, Germany; Crop Functional Genomics, Institute of Crop Science and Resource Conservation (INRES), University of Bonn, 53115 Bonn, Germany; Crop Functional Genomics, Institute of Crop Science and Resource Conservation (INRES), University of Bonn, 53115 Bonn, Germany; Crop Bioinformatics, Institute of Crop Science and Resource Conservation (INRES), University of Bonn, 53115 Bonn, Germany

## Abstract

**Motivation:**

Insertional mutagenesis allows for the creation of loss-of-function mutations on a genome-wide scale. In theory, every gene can be ‘knocked out’ via the insertion of an additional DNA sequence. Resources of sequence-indexed mutants of plant and animal model organisms are instrumental for functional genomics studies. Such repositories significantly speed up the acquisition of interesting genotypes and allow for the validation of hypotheses regarding phenotypic consequences in reverse genetics. To create such resources, comprehensive sequencing of flanking sequence tags using protocols such as Mutant-seq requires various downstream computational tasks, and these need to be performed in an efficient and reproducible manner.

**Results:**

Here, we present MuWU, an automated **Mu**tant*-*seq **w**orkflow **u**tility initially created for the identification of *Mutator* insertion sites of the *BonnMu* resource, representing a reverse genetics mutant collection for functional genetics in maize (*Zea mays*). MuWU functions as a fast, one-stop downstream processing pipeline of Mutant-seq reads. It takes care of all complex bioinformatic tasks, such as identifying tagged genes and differentiating between germinal and somatic mutations/insertions. Furthermore, MuWU automatically assigns insertions to the corresponding mutated seed stocks. We discuss the implementation and how parameters can easily be adapted to use MuWU for other species/transposable elements.

**Availability and implementation:**

MuWU is a Snakemake-based workflow and freely available at https://github.com/tgstoecker/MuWU.

**Supplementary information:**

Supplementary data are available at *Bioinformatics* online.

## 1 Introduction

Both in forward and reverse genetic studies, sequence-indexed insertional libraries constitute essential resources for researchers around the globe. Notable examples are transgenic T-DNA insertion lines in Arabidopsis (Alonso *et al.*, 2003) or several mutant collections based on the use of transposable elements (TEs) in rice (Hirochika *et al.*, 2004) and maize (Liang *et al.*, 2019; Marcon *et al.*, 2020; McCarty *et al.*, 2005). For efficiency, when screening large mutant collections, targeted sequencing approaches such as Mutant-seq (Mu-seq; McCarty *et al.*, 2013; Supplementary Fig. S1) are used.

Mutagenized families are pooled according to a grid design (Supplementary Fig. S2; McCarty *et al.*, 2013; Urbański *et al.*, 2012). Mu-seq can take this one step further by distinguishing between somatic and germinal (heritable) mutations. If row and column pools are taken from independent somatic cell lineages, heritable insertions have to appear in both axes of the grid and can thus be singled out for further analyses. To our knowledge, MuWU is the first openly available tool for the analysis of insertional libraries generated using a Mu-seq approach. MuWU efficiently combines the necessary bioinformatics analyses to enable the benefits of the Mu-seq approach: filtering of pre-existing insertion sites as well as somatic insertions. In contrast to other approaches, MuWU does not require paired-end sequencing.

MuWU was created as a solution to the recurring processing of *Mu* insertional mutagenesis sequencing libraries in maize as part of the newly created and expanding *BonnMu* resource (Marcon *et al.*, 2020), the first European mutant resource of its kind. *BonnMu* insertions are continuously being integrated into the MaizeGDB.org genome browser (https://www.maizegdb.org; Portwood *et al.*, 2019).

## 2 Software description

### 2.1 Input files and data preparation

All software and dependencies are either installed at runtime or run inside a singularity container allowing for completely automated detection and annotation of insertion sites. The grid design of a Mu-seq experiment results in 2*n* samples, with the size *n* in our experiments usually being 24 pools of 24 F2 families each. This results in the sequencing of 576 families in 48 sequencing libraries (24 row and 24 column pools). In addition to sequencing reads, MuWU requires genome sequence FASTA file as well as a suitable annotation file. Also, a library-specific stock matrix table should be supplied indicating the position of each mutagenized maize family in the row x column grid design to infer germinal insertions (Supplementary Fig. S2).

The first steps of MuWU deal with quality control and alignment of the reads to the reference genome sequence. Most notable are the removal of transposon terminal inverted repeat (TIR) sequences and adapters from both ends of the raw Mu-seq reads. A summary of all statistics is generated in one HTML report.

### 2.2 Annotation procedure

Insertion sites are identified with our Python tool (insertions.py) which takes advantage of the TE-specific target site duplication (TSD) at the insertion flanking region. At this first step, at least two sequencing reads at both sides of the TSD are required to support the insertion. In a second step, germinal insertion sites of one row and one column pool of the grid layout are filtered to allow assignment of a distinct mutagenized family. Thus, heritable mutations are identified by keeping insertions with shared genomic coordinates in only one row and one column sample each. Parallel implementation of both steps of the algorithm allows us to circumvent this initial computational bottleneck - with a 1 thread per sample granularity reducing runtime significantly.

In MuWU’s subsequent annotation, we analyze insertion sites inside or near gene models. By identifying the specific combination of row and column pool for each germinal insertion event, we can perform a matrix lookup in the library-specific seed stock table and add stock information to the output. The final outputs are a table of germinal insertions, their read coverage and corresponding stock, as well as id, gene lengths and coordinates of the genes the insertions were assigned to, and a similar table for all insertions. The complete workflow is shown in Supplementary Figure S3.

## 3 Conclusion

MuWU is an efficient workflow solution reducing the bioinformatics steps of the *BonnMu* database resource to a one-command job finished in <1 h per library (Intel(R) Xeon(R) CPU E5-2690 v4@ 2.60 GHz; 24 cores). It implements the bioinformatics part of Mu-seq, an improved strategy to distinguish between germinal and somatic insertions, combining the advantages of an experimental layout in grid design with the analysis of TSDs (Liang *et al.*, 2019; McCarty *et al.*, 2013). Automation of library annotation is essential for reproducibility and consistency as we continue to expand our database effort with upcoming libraries.

While MuWU is not the only bioinformatics tool for TE insertion detection, it is to our knowledge the only openly available tool suitable for Mu-seq data and, in contrast to other tools for TE insertion detection, works with single-end sequencing as it does not rely on the analysis of discordant read pairs. MuWU is not specific for *Mutator* insertions or maize but can detect any insertion event that causes TSDs. The user can configure TE-specific and adapter sequences, TSD length and read support threshold. We have implemented a secondary mode (‘GENERIC’) which does not require an experimental design that allows inferring germinal insertion events and is thus more widely applicable. As the workflow is built on Snakemake (Köster and Rahmann, 2012), the addition of further analyses to the automatic handling of Mu-seq libraries allows for modular expansion of its current state.

## Supplementary Material

btab679_Supplementary_Data
