# Supplementary material for: MuWU: Mutant-seq library analysis and annotation
Source: Bioinformatics. 2021 Sep 29;38(3):837–8. doi: 10.1093/bioinformatics/btab679 (PMC8756183; doi:10.1093/bioinformatics/btab679)
Supplement: btab679_Supplementary_Data [file btab679_supplementary_data.docx]

**
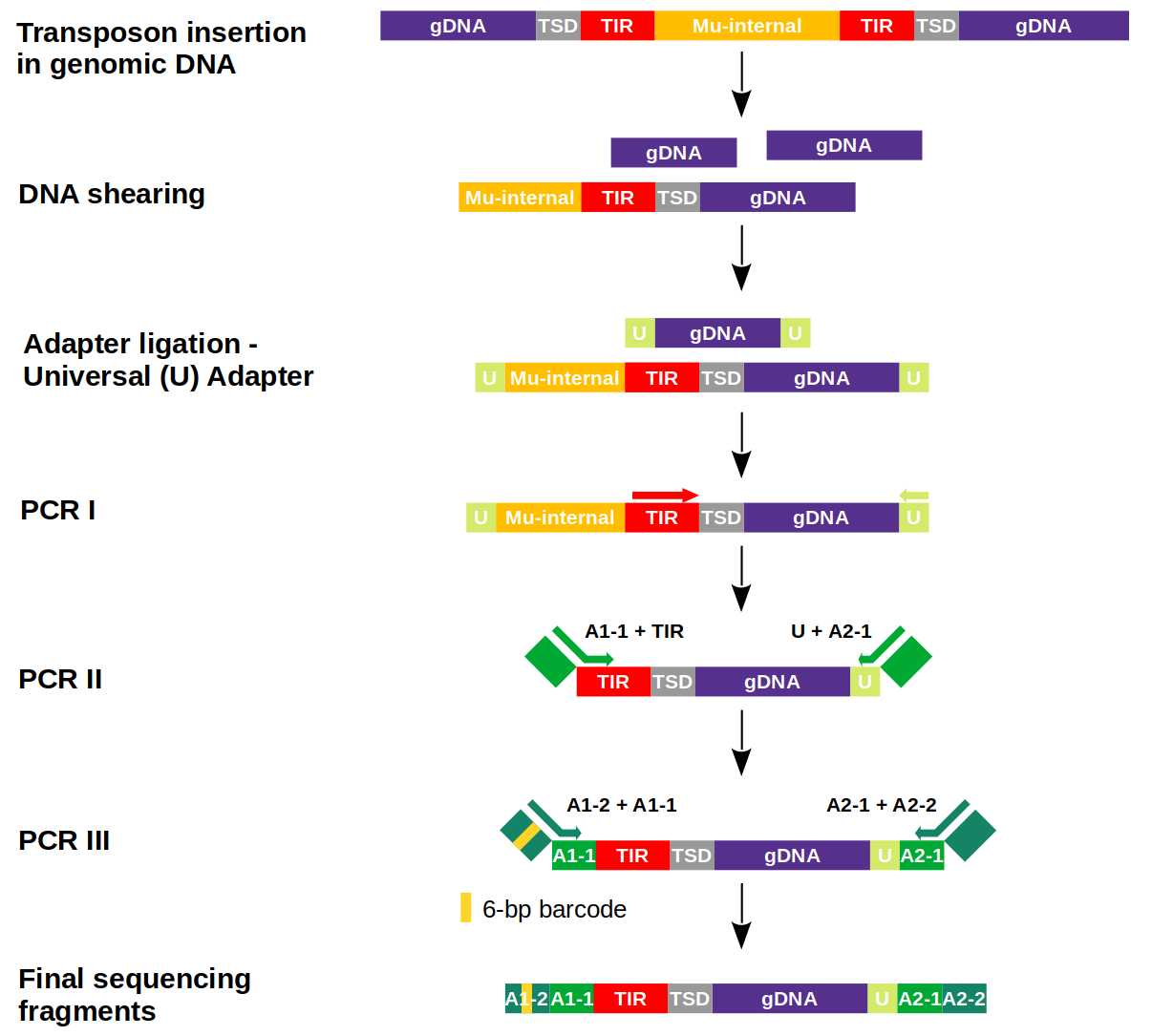
**

**Supplemental Figure 1.**

Mu-seq library construction (Marcon et al., 2020) starts with genomic DNA isolation from pools of 576 maize families, followed by DNA shearing and ligation of a universal (U) adapter. PCR I enriches Mu-TIR flanking DNA. PCR II+III incorporate Illumina sequencing adapters A1 and A2 respectively and a 6-bp barcode enabling multiplexing of 48 pools in a single library.

**
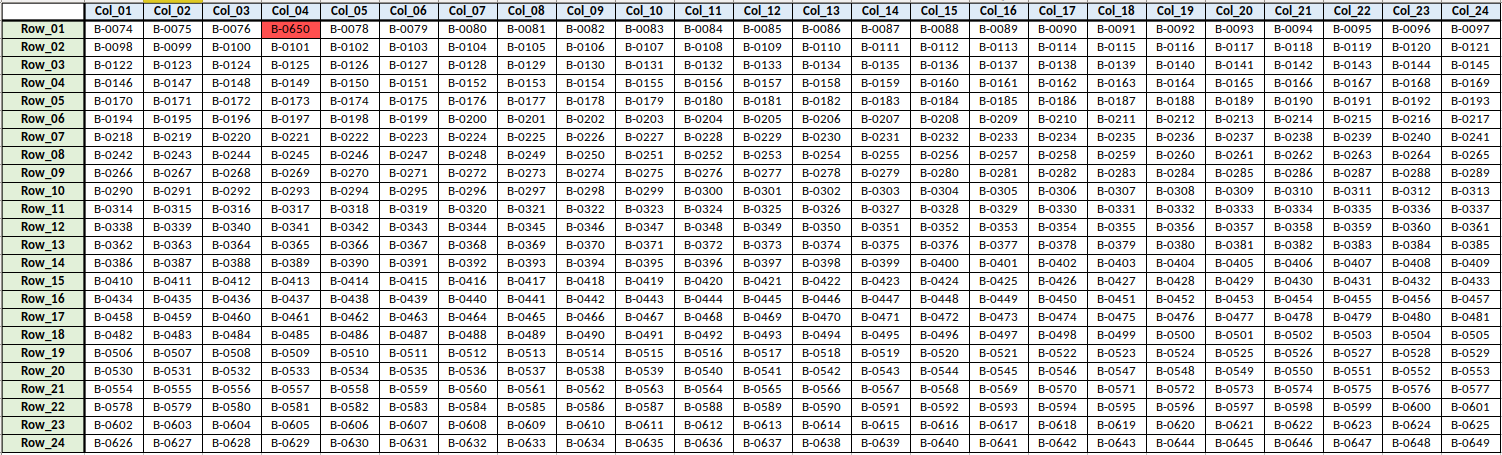
**

**Supplemental Figure 2.**

Pooling design of mutagenized maize families according to a grid. Library specific seed stock matrixes are used by MuWU to annotate every germinal insertion by checking the specific combination of row and column pool for each insertion event.


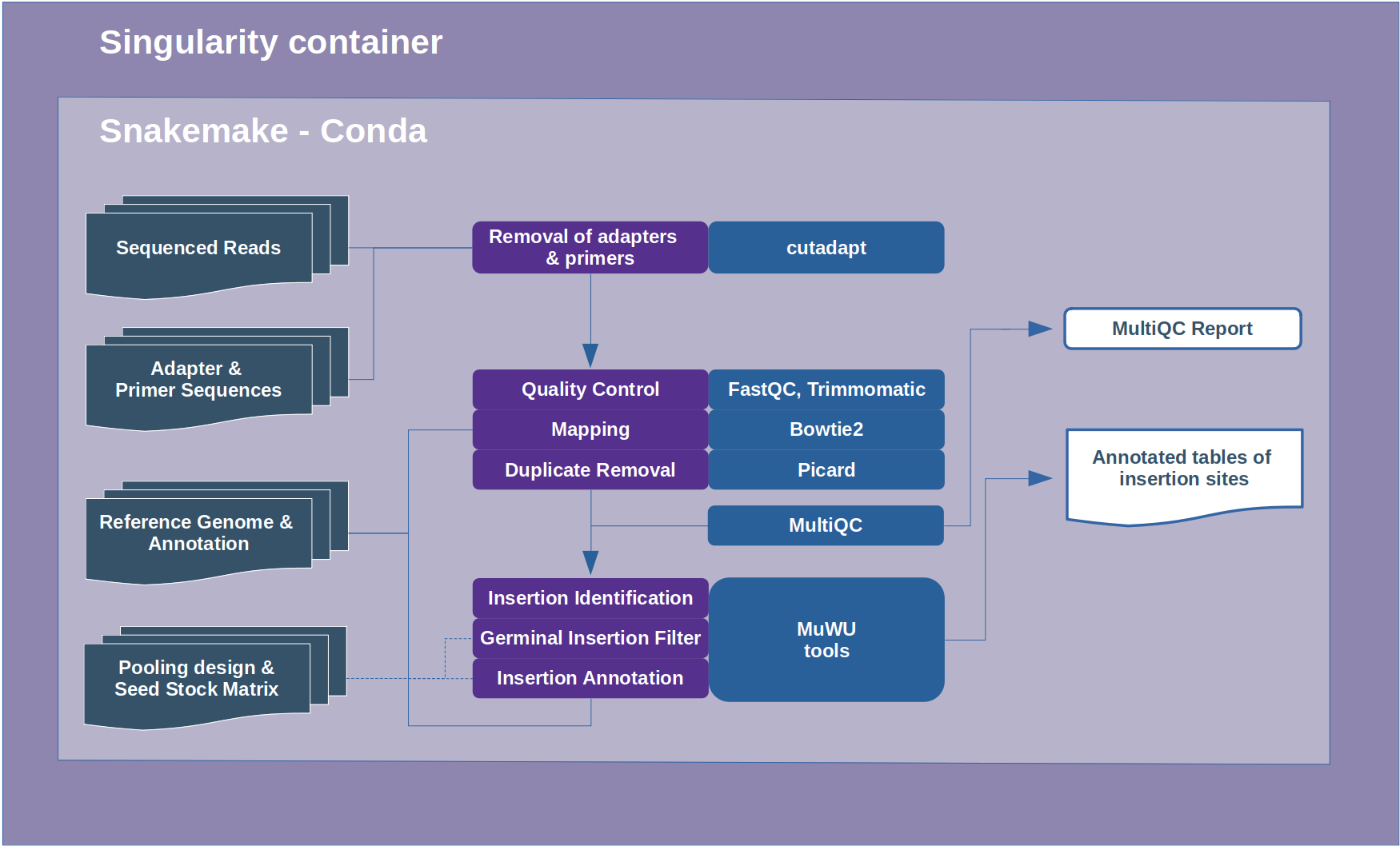


**Supplemental Figure 3.**

Workflow diagram for MuWU.

Required input files:

- Reference Genome: un-/gzipped FASTA or GenBank (.gbff, .dat) - file location or URL
- Annotation: un-/gzipped GFF3, GTF or GenBank (.gbff, .dat) format - file location or URL
- DNA sequencing reads: un-/gzipped single/paired end FASTQ files
- Primer/Adapter sequences: either pasted into config.yaml or provided as separate FASTA file/s
- GRID method: Pooling Design/Stock Matrix table: .xlsx file (see Supplemental Figure 2.); GENERIC method: samples.tsv table listing samples

Supplemental Information 1.

MuWU parameters.

All options of the workflow including which of the two methods to chose, input files and parameters (e.g. thread usage per step) can be set in the config/config.yaml.
The config.yaml includes detailed explanations of all options with the default parameters representing our work in the BonnMu project.
The most important parameters - those controlling MuWU's custom tools - are briefly discussed in the following.
Besides suitable handling of primers/adapters via trimming using cutadapt & trimmomatic the config.yaml also allows to set:

"approach", "overlap_size", "overlap_support" and "extension"

- "approach" can be set to either GRID or GENERIC, defining whether to use a 2d pooling approach to also identify germinal insertions or to perform a GENERIC insertion detection without a germinal/somatic distinction.
- With "overlap_size" the user can change the length of the overlap region (start/end of reads) used inside identification algorithm. This should be set equal to the length of TSD of the transposon being investigated (default is 9 = Mutator transposons).
- "overlap_support" controls the minimum amount of support needed to call an insertion (default is 2). With this default it would necessitate at least two reads that end & 2 reads which start with the overlap (TSD sequence at particular locus).
- Using "extension", upstream or downstream bases (equal to integer value) can be added to the gene models of the annotation to include UTRs, regulatory regions, promoter sequences etc.. This is helpful since insertions outside of gene models can otherwise not be annotated and thus allows for easier association of these regions with the gene at the particular locus.

Supplemental Table 1.

Complete list of software and respective version

| **Software** | **Version** |
| --- | --- |
| bioconductor-iranges | 2.26.0 |
| biopython | 1.78 |
| bowtie2 | 2.4.1 |
| GNU coreutils | 8.31 |
| cutadapt | 2.10 |
| fastqc | 0.11.9 |
| gffread | 0.12.1 |
| grep | 3.4 |
| mamba | 0.14.1 |
| multiqc | 1.10.1 |
| numpy | 1.16.4 |
| pandas | 0.25.0 |
| picard | 2.22.1 |
| pigz | 2.3.4 |
| python | >=3.6 |
| r-base | 4.1.0 |
| r-dplyr | 1.0.6 |
| r-fuzzyjoin | 0.1.6 |
| r-readxl | 1.3.1 |
| r-stringr | 1.4.0 |
| samtools | 1.10 |
| seqkit | 0.16.1 |
| snakemake | 6.4.1 |
| snakemake-wrapper-utils | 0.2.0 |
| tbb | 2020.2 |
| trimmomatic | 0.36 |
